# Dr. Russell Davies (1914–1991): Pioneer of theatre recovery and of anaesthetics in Yugoslavia

**DOI:** 10.1177/09677720241230687

**Published:** 2024-03-11

**Authors:** Holly Elizabeth Webster, Maxwell John Cooper

**Affiliations:** 12190 Brighton and Sussex Medical School, Brighton, United Kingdom of Great Britain and Northern Ireland

**Keywords:** Anaesthesia, East Grinstead, England, Guinea Pig Club, Queen Victoria Hospital, recovery ward, Russell Davies, Sussex, theatre recovery, Yugoslavia

## Abstract

Dr. Russell Davies is a largely forgotten pioneer of both post-operative theatre recovery but also a key figure in the establishment of anaesthetics services in Yugoslavia in the late 1940s. Davies spent the majority of his career working as an anaesthetist at Queen Victoria Hospital, East Grinstead, Sussex, England, later being promoted to the head anaesthetist role. Davies set up one of the first recovery wards in the United Kingdom at Queen Victoria Hospital, the ward being named after him in 1989. Here he became a founding member of the Guinea Pig Club, alongside Dr. Archibald McIndoe. The Guinea Pig Club was founded in 1941 to support airmen in the Second World War undergoing plastic surgery at Queen Victoria Hospital. Davies was crucial to the pastoral care of the Club, providing clinical care and guiding members over access to pensions they would have previously been denied. Little is recognised of Davies's achievement of establishing anaesthetics services in Yugoslavia. Davies and his contributions have been largely overlooked. Davies should be considered one of the foremost British anaesthetists of the 20th century.

## Family and education

Russell Maddox Davies was born on 6^th^ June 1914 in Ponthir, Newport, Monmouthshire in Wales.^1^ He was born to Oswald (1875–1933) and Alice May Davies (1882–1959) and had an older brother, Philip Maddox (1910).^2^ His parents married in Pontypool, Monmouthshire in 1908 before moving to the centre of Newport, where they lived and raised their children.^2^

Davies was educated at Newport High School for Boys, King's College, London and Westminster Hospital Medical School.^3^ He graduated as a doctor in 1939^4^ and was awarded the Diploma in Anaesthesia (D.A) in 1941.^5^ In 1953 Davies was made a Fellow of the Faculty of Anaesthetists (F. F. A. R. C. S).^6^ The Faculty had been founded in 1948 within the Royal College of Surgeons of England (with the status of Fellow replacing the previous Diploma in Anaesthetics (D.A)). The Royal College of Anaesthetists was established as an independent institution in 1992.^7^

Davies first married Joan E. Cooper in 1941 with whom he had two daughters, Jane and Penny.^8^ He later married his second wife, Kathleen J. Packer, in Winchester, Hampshire in 1983.^2,9^ Latterly known as Dr. Kate Davies, she worked as an anaesthetist in Winchester Hospital.^2,10^ Following his retirement, the couple relocated to Winchester, where he spent his later years.^1^

## Early career (1941–1953)

Davies began his clinical career as a resident anaesthetist at Westminster Hospital, until he signed up for the Emergency Medical Service following the outbreak of the Second World War. He was posted to the Queen Victoria Hospital, East Grinstead in 1941.^9^ His initial appointment was as assistant to Dr. John Hunter (died 1953), head anaesthetist under Dr. Archibald McIndoe. Davies became a consultant at Queen Victoria Hospital in 1946 and subsequently was appointed head anaesthetist following Dr. John Hunter's death.^1^

Davies was fiercely proud of the National Health Service and strongly supported its values, so much so that he turned down the opportunity to become McIndoe's head anaesthetist following Hunter's death. Davies felt that he ‘could not have served two masters’, one being the state and the other private medicine.^11^ Davies remained a consultant at Queen Victoria Hospital until his retirement in 1974.^1^

## The Guinea Pig Club 1941

Davies is regarded to be a founding member of the Guinea Pig Club.^1^ The Guinea Pig Club was founded in 1941 to support wounded airmen undergoing plastic surgery at Queen Victoria Hospital. The Club gained its name from the members viewing themselves as the guinea pigs for the pioneering plastic surgery by McIndoe and was initially a social club.^12^ The Club grew from 39 members to 649 by the end of the Second World War and provided support for the members in every aspect of their lives.^13,14^ Davies was described as having a ‘quiet demeanour’, especially in light of his close working relationship with McIndoe. This was in contrast to McIndoe who was known for being a ‘larger-than-life personality’.^9,10^ Davies had a ‘steely determination’ and fought for whatever he believed in, such as advancing financial support for the Guinea Pig Club members.^1,9,10^

Davies fought to establish funding from the Royal Air Force Benevolent Fund, alongside driving change in the attitude of the government’s Pensions Department to allow the burned airmen to claim pensions as other wounded soldiers were entitled too. Initially, none of the members were awarded 100% pensions but Davies liaised with the Pensions Department for a more sympathetic approach. This changed the lives of the injured airmen: they were now entitled to receive a pension, and recognised among war wounded and disabled.^1,15^ Davies was described as a ‘caring, unselfish benefactor who was never afraid to champion their cause’ by Guinea Pig Club members.^11^

## Founding of the recovery ward in East Grinstead 1946

The first recovery ward in the world was reported to have been opened in Massachusetts, United States in 1873 although it did not resemble Davies's recovery ward nor a modern recovery ward: the admission duration was short and the room lacked equipment.^16^ Davies himself was aware of a recovery ward opened in Minnesota, United States in 1942, providing one bed per theatre and open from 9 a.m. to 5 p.m.^17^ Recovery wards became more widely accepted in the United States during the Second World War whereas the concept of a recovery ward did not gain traction in the United Kingdom until 1961, with a programme of building new hospitals. It was only in the 1960s and 1970s that recovery wards became widely available across the United Kingdom, rather than being previously dependent on local hospital services. Prior to the 1960s and 1970s only a number of hospitals scattered across the United Kingdom had built recovery wards.^16^

Davies's most renowned work was his research into and subsequently opening the first theatre recovery ward in the United Kingdom at Queen Victoria Hospital.^1,17^ The unit first opened in 1946, with 10 beds and 10 rooms, next to theatres and radiology and with a dedicated nursing staff. The design of the ward is still evident today in every recovery ward in the United Kingdom, each bedspace containing piped oxygen, suction, a call bell and hand washing facilities. All of which is still present in modern recovery wards.^17^ The ward was immediately successful, in the first 11 years it welcomed just under 30,000 patients, with only nine deaths. The mortality rate was more than halved in comparison to sending patients immediately back to the ward following surgery.^18^ It was noted that Davies ‘always took his lunch there instead of going to the “Doctor's Mess” so he could look after any postoperative problems’ showing his commitment to the success of the unit and his patients’ well-being.^17^

Davies set out principles of the recovery ward, a majority of which are still adhered to today in modern recovery wards. These principles included: the staffing of the ward 24 h a day; all patients should come to this ward following a general anaesthetic, ‘the required level of nursing skill must be high’ and the nursing staff never to be moved in blocks from the ward; the ward must be close to theatres; available medical care and equipment at all times and the retention of the patient for as long as it will benefit them.^18^

**Figure 1.**
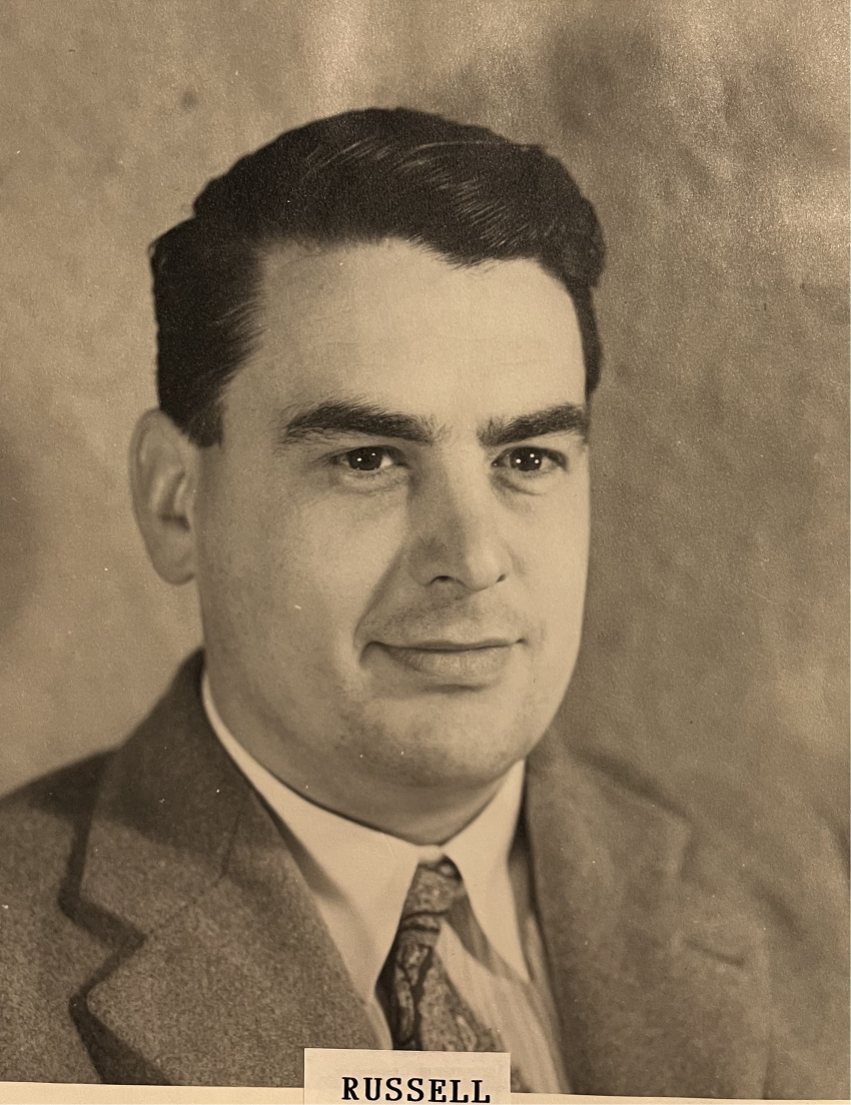
Dr. Russell Davies (taken in the 1940s). Image courtesy of Mr. Robert Marchant, East Grinstead Museum and Queen Victoria Hospital.

Before the establishment of recovery wards patients were often subjected to long journeys back to hospital wards immediately following surgery, increasing the risk of respiratory complications. Commonly this was obstruction or inadequate respiratory effort, arising before the patient had even reached the wards.^18^ This increased mortality rates before the establishment of the recovery ward, where the journey was short and complications could be dealt with by trained staff and resuscitation equipment.^18^ ‘Davies made a lasting contribution to medical practice when he campaigned for the establishment of the recovery ward’ reducing patient mortality and setting out principles still employed today (Figure 1).^10^

## Developing anaesthesia in Yugoslavia 1946

Davies played a central role in the establishment of anaesthetics services in Yugoslavia. This started in 1946 through the United Nations Relief and Rehabilitation Administration (UNRRA)^19^. This was also the result of the establishment of a plastic surgery unit there by Dr. Harold Gillies (1882–1960) with the support of the British Army. Gillies is considered as a pioneer of plastic surgery. McIndoe was a first cousin, once removed, of Gillies.^19^

Yugoslavia had been devastated by war and occupation; despite many casualties it was largely lacking in anaesthetics knowledge or training, with only local anaesthetic previously being widely known and used.^20^ Evidence of the lack of provision and trust in Yugoslavia towards anaesthetics at the time is laid bare by the following description ‘even when we have anaesthetics the girls often refuse to take them’, describing young women in Yugoslavia during the Second World War undergoing very painful operations by Evelyn Waugh, an author and former soldier who was posted to Yugoslavia in 1944.^17,21^

Davies first began his work in Yugoslavia in 1946, sharing anaesthetics knowledge through demonstrations of the superior value of general over local anaesthesia in certain cases for the patient's recovery, spreading the word of modern anaesthesia and aiding the acceptance of general anaesthesia.^20^ He did this alongside Dr. Patrick Shackleton (1904–1977), a fellow anaesthetist.^22^ Russell would travel to a new hospital with equipment and staff to set up an anaesthetic department, often in response to formal invitation. Russell trained local anaesthetists and supplied literature, even venturing to attempt to set up a nitrous oxide supply in Yugoslavia.^20^

Davies left Yugoslavia after seven months and was not replaced because UNRRA withdrew their mission although he remained in contact with Yugoslavian anaesthetists, later visiting to present papers at meetings.^20^ He suggested the initiation of a journal and registrar's prize for Yugoslavia at the World Conference of Anaesthesiologists in London in 1968.^20^ This led to the creation of the Bettini medal (Bettini being believed to have been the first Yugoslavian to have administered general anaesthesia in 1847). Shackleton and Davies were the first recipients of the medal in 1973 for services to Yugoslavian Anaesthesia.^3,20^ Davies and Shackleton are considered to be the fathers of modern anaesthesia in Yugoslavia: ‘they gathered around them a group of our doctors with whom they shared their knowledge and experience. And from this little group of doctors, modern anaesthesia spread to all parts of the country’ by a Yugoslavian professor of anaesthesia.^23^

## Later life (1954–1967)

Davies spent time as a guest lecturer in America, visiting Chicago twice, first spending six months as a Fulbright Exchange Professor at the University of Illinois in 1954 and later three months at Northwestern University, Illinois in 1967.^1^ Davies also taught about anaesthesia for plastic surgery for the Royal College of Surgeons of England in 1960.^24^

Davies was also involved in the education of junior anaesthetics staff, medical students, international doctors for post-graduate training, operating department assistants (ODA) and nurses. The hosting of international doctors training being unusual outside of London hospitals at the time.^3^ Davies is fondly remembered by a former chief ODA at Queen Victoria Hospital, described as being ‘well respected, not only by his fellow consultants, but by all the staff he had contact with’ and ‘always ready to spend time teaching and talking to his juniors’.^25^

## Academic contributions

**Figure 2.**
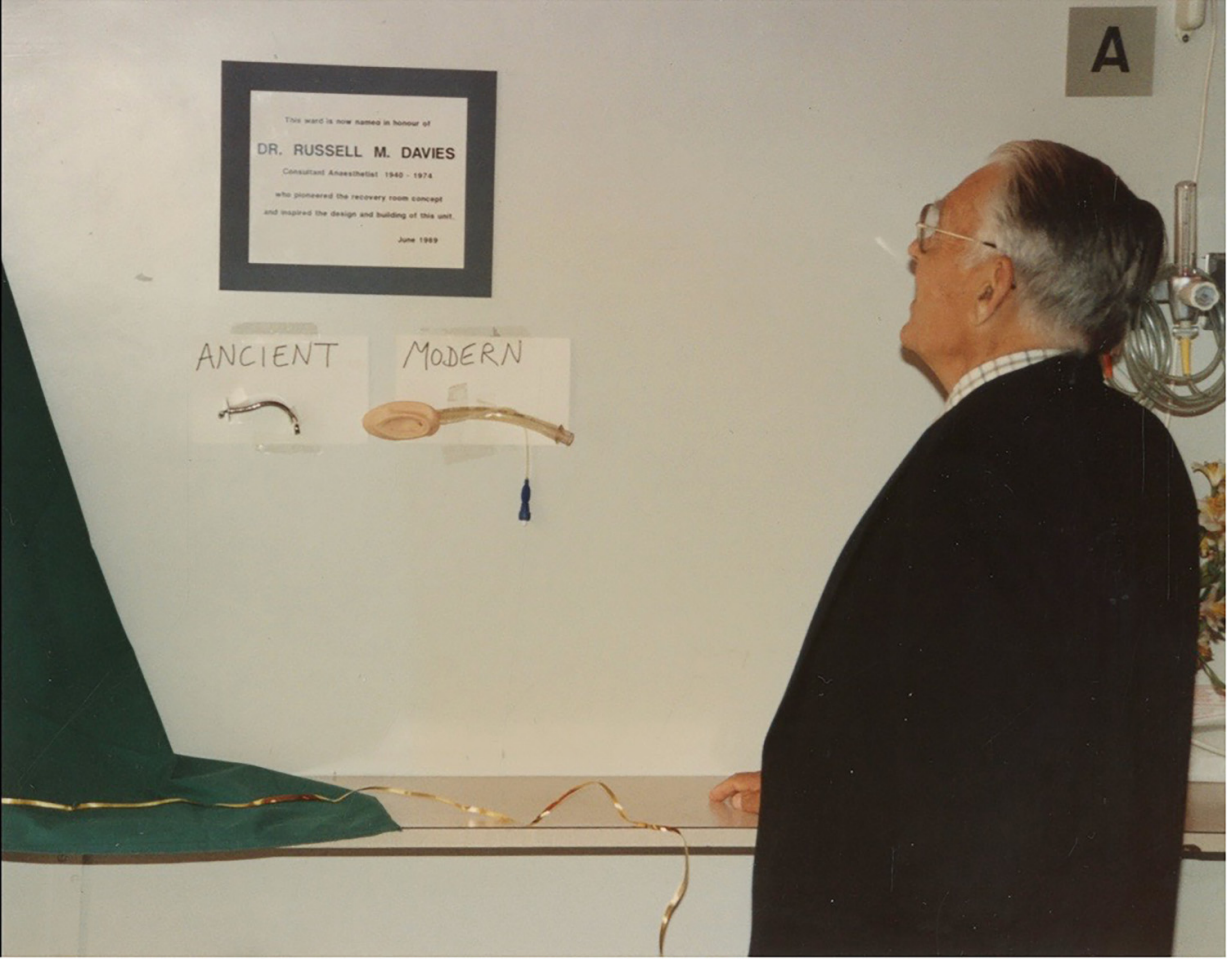
Dr. Russell Davies at the opening of the RDU, 1989. Image courtesy of the East Grinstead Museum.

Davies completed 30 publications in his working lifetime, 28 of those on anaesthetics matters and two on specialist plastic surgery equipment.^3^ Alongside his academic endeavours and establishment of theatre recovery in the United Kingdom, he also wrote about matters such as endotracheal tubes, aiding the development of newer modern tubes as opposed to the older metal versions. In addition, he wrote about hypotensive anaesthesia, a method in part pioneered at East Grinstead by Dr. Hale Enderby, alongside Davies (Figure 2).^26,27^

Davies wrote about the hazards of hypotensive anaesthesia, emphasising the level of training required for the method and the importance of collaboration between anaesthetist, surgeon and recovery ward nurse, as well as patient factors. This again highlighted Russell's drive to ensure favourable clinical outcomes for the patient.^26^ Davies was a founding trustee of the East Grinstead Medical Research Trust in 1959, based at the Blond McIndoe Centre, with aims to further research into burns, plastic surgery and pain.^9,15,28^ Russell was invited to present his work with McIndoe, delivering the ninth McIndoe Memorial Lecture entitled ‘Relationships’ in 1976 to the Royal College of Surgeons of England.^9,29^

Davies, alongside Dr. Stuart Laird, founded the Association of Burns and Reconstructive Anaesthesia.^27^ Laird was a consultant anaesthetist at St Lawrence Hospital, Chepstow, Monmouthshire Wales, where he researched analgesia in burns.^30,31^ The association awarded an annual prize, the Russell Davies and Stuart Laird Prize, to an association trainee for a paper on both anaesthetics and plastic surgery or burns.^30^ The association was founded in 1971 following a symposium organised by Russell and Laird. It was the first of its kind, based on the role of anaesthetics in burns patients and was attended by not only anaesthetists but surgeons also. This fostered the idea of a multi-disciplinary approach (MDT), especially towards the inclusion of anaesthetists in the MDT, to the treatment of these patients.^30,32^ Unfortunately, the association is no longer active, the prize having last been awarded in 2005.^32^

## Retirement in Hampshire (1974–1991)

Davies was a keen volunteer, working as an ambulance car driver from 1974 to 1984 and volunteering at the Winchester Archeology Society, where he became an expert in bell founding.^1^ Davies was also an enthusiast of Exmoor, creating a comprehensive bibliography of books about Exmoor, alongside his wife, Kate.^28^

Perhaps his most important volunteering work was that which he maintained at the Guinea Pig Club, he worked not only as part of the crucial medical team but also continued to provide pastoral care. He continued to aid the Guinea Pig Club Members to organise disability benefits for the remainder of his life, something he had fought for and established over 40 years prior.^1,15^

## Death and legacy

**Figure 3.**
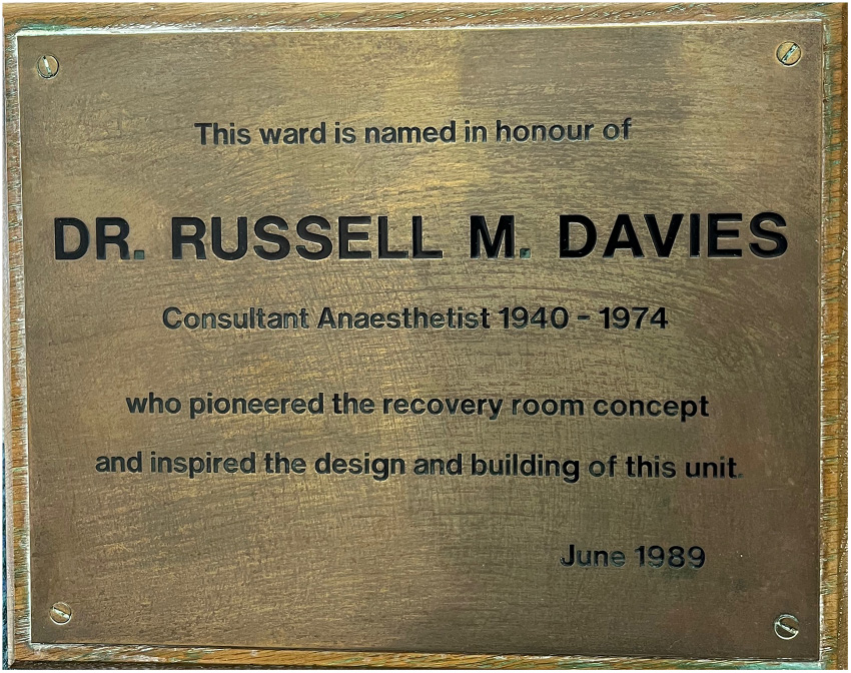
The Russell Davies Unit plaque, presented to Davies in 1989. Image by Holly Webster, 13th October 2023 Queen Victoria Hospital.

Davies was described as the ‘greatest friend the Guinea pig club ever had’ in a tribute to Davies in the Guinea Pig Club magazine and his work with disability pensions was described as ‘legendary’.^11,28^ The theatre recovery ward Russell set up was aptly named the Russell Davies Unit in 1989. Davies was present for the opening, for which he was ‘surprised and delighted that it had happened in his lifetime’ (Figure 3).^1,17^

Davies was invited to a meeting in Yugoslavia in 1988 but was too frail to attend so instead a colleague, Dr. John Zorab, attended and received a specially engraved medal to present to Davies on his return.^23^ The medal recognised Davies's achievements in Yugoslavia; indeed he is still regarded as their father of modern anaesthesia.^23^ Davies's last engagement with the Guinea Pig Club was their 50th-anniversary dinner in 1991, he was warmly welcomed despite being very frail.^8^

This is keenly demonstrated by the 30 members of the Guinea Pig Club and Queen Victoria Hospital representatives who attended Davies's funeral following his death. He died on the 13th of October 1991 in Winchester Hospital, aged 77. His funeral was held in Salisbury Crematorium on 22nd October 1991.^8,11^ Davies is survived by his legacy of pioneering theatre recovery wards, developing Yugoslavian anaesthetics services, and enhancing anaesthesia services at Queen Victoria Hospital alongside his work with the Guinea Pig Club. Russell is survived by his wife Kate and his two daughters, Jane and Penny.

Davies's life-long work with and dedication to the Guinea Pig Club aided many members both medically and financially. Every patient who passes through a recovery ward following surgery also benefits from Davies's design and ideas. Davies's principles and recovery ward layout are still very clearly seen today in any recovery ward.^18^ Queen Victoria Hospital maintains a standard of excellence in anaesthesia as well as plastic surgery, no doubt contributed too by Davies.^33^ Finally, Davies is still credited with founding modern anaesthesia in Yugoslavia, sharing knowledge and skills across the country to revolutionise anaesthesia.^24^

## Conclusion

Davies is remembered for his quiet, unassuming but determined character, for his longstanding commitment to the Guinea Pig Club and for advancing anaesthesia recovery in the United Kingdom.^15^ He has largely faded from popular medical memory, being overshadowed by larger figures, particularly McIndoe, with both men closely involved with the Guinea Pig Club and Queen Victoria Hospital.

Davies did not, on his own, invent recovery wards or establish anaesthesia in Yugoslavia, but he played a pivotal role in the development of both and those influences still bring benefits. Sadly many of the beneficiaries of Davies's work are unaware of his contributions. This article seeks to record the achievements of Dr. Russell Davies.
